# Variant detection and the Autism sequencing project

**DOI:** 10.1186/1471-2105-12-S11-A4

**Published:** 2011-11-21

**Authors:** Orion Buske, Misko Dzamba, Justin Foong, Lynette Lau, Marc Fiume, Christian Marshall, Susan Walker, Aparna Prasad, Michael Brudno

**Affiliations:** 1 Department of Computer Science, University of Toronto, Toronto, Canada; 2 The Centre for Applied Genomics, The Hospital for Sick Children, Toronto, Canada; 3 Donnelly Centre, University of Toronto, Toronto, Canada

## Background

Early detection of autism can improve the quality of life of affected individuals [1]. Qualitative screening methods continue to improve, but still suffer from low sensitivity despite increasing specificity [2,3]. In collaboration with the Hospital for Sick Children, we are sequencing the exomes of 1 000 individuals with autism in order to discover genetic variants associated with the disorder. Discovery of associated variants can lead to earlier diagnosis and treatment.

## Materials and methods

We will present our current sequencing and analysis pipeline, from SureSelect exome capture and SOLiD sequencing through Sanger validation of predicted harmful variants, along with tools we have developed for color-space-aware alignment, variant detection, and visualization of next-generation sequencing data.

Color-space sequencing provides a tradeoff between enhanced ability to distinguish Single Nucleotide Variants (SNVs) from sequencing errors at the price of a higher sequencing error rate versus traditional letter-space sequencing. This technology has the potential to provide higher accuracy at lower cost, but opens new computational challenges that need to be addressed.

## Results and conclusions

We have sequenced over 70 individuals so far at approximately 30x mean coverage, have found and validated several interesting non-synonymous Single Nucleotide Variants (SNVs), and have identified a number of potential de novo non-synonymous mutations. After filtering, we are identifying an average of over 17 000 non-synonymous SNVs per individual, of which over 11 000 are novel to dbSNP. We also find that support from both strands is more informative than total depth of coverage for predicting SNVs from high-throughput sequencing data. This is of considerable importance in exome capture data, since only a small region at each probe captures sequence from both strands.
